# Evaluation of HPV Infection and Smoking Status Impacts on Cell Proliferation in Epithelial Layers of Cervical Neoplasia

**DOI:** 10.1371/journal.pone.0107088

**Published:** 2014-09-11

**Authors:** Martial Guillaud, Timon P. H. Buys, Anita Carraro, Jagoda Korbelik, Michele Follen, Michael Scheurer, Karen Adler Storthz, Dirk van Niekerk, Calum E. MacAulay

**Affiliations:** 1 Department of Integrative Oncology, British Columbia Cancer Research Centre, Vancouver, British Columbia, Canada; 2 Department of Obstetrics and Gynecology, Paul L. Foster School of Medicine, Texas Tech University Health Sciences Center at El Paso, El Paso, Texas, United States of America; 3 Department of Pediatrics, Dan L. Duncan Cancer Center, Baylor College of Medicine, Houston, Texas, United States of America; 4 Department of Diagnostic Sciences, School of Dentistry, University of Texas Health Science Center, Houston, Texas, United States of America; 5 Department of Pathology, British Columbia Cancer Agency, Vancouver, British Columbia, Canada; Georgetown University, United States of America

## Abstract

Accurate cervical intra-epithelial neoplasia (CIN) lesion grading is needed for effective patient management. We applied computer-assisted scanning and analytic approaches to immuno-stained CIN lesion sections to more accurately delineate disease states and decipher cell proliferation impacts from HPV and smoking within individual epithelial layers. A patient cohort undergoing cervical screening was identified (n = 196) and biopsies of varying disease grades and with intact basement membranes and epithelial layers were obtained (n = 261). Specimens were sectioned, stained (Mib1), and scanned using a high-resolution imaging system. We achieved semi-automated delineation of proliferation status and epithelial cell layers using Otsu segmentation, manual image review, Voronoi tessellation, and immuno-staining. Data were interrogated against known status for HPV infection, smoking, and disease grade. We observed increased cell proliferation and decreased epithelial thickness with increased disease grade (when analyzing the epithelium at full thickness). Analysis within individual cell layers showed a ≥50% increase in cell proliferation for CIN2 vs. CIN1 lesions in higher epithelial layers (with minimal differences seen in basal/parabasal layers). Higher rates of proliferation for HPV-positive vs. -negative cases were seen in epithelial layers beyond the basal/parabasal layers in normal and CIN1 tissues. Comparing smokers vs. non-smokers, we observed increased cell proliferation in parabasal (low and high grade lesions) and basal layers (high grade only). In sum, we report CIN grade-specific differences in cell proliferation within individual epithelial layers. We also show HPV and smoking impacts on cell layer-specific proliferation. Our findings yield insight into CIN progression biology and demonstrate that rigorous, semi-automated imaging of histopathological specimens may be applied to improve disease grading accuracy.

## Introduction

Predicting outcomes for cervical intra-epithelial neoplasia (CIN) lesions remains a complex challenge. Some lesions progress to later disease stages while others do not, meaning some patients experience risks and costs of treatment unnecessarily. Further, HPV infection status for normal and early CIN tissues may be insufficient for stratifying progression risk. New tests are needed to accurately stratify patients presenting with CIN and to reduce the number of women treated unnecessarily for high grade squamous intraepithelial lesions (HGSILs).

Multiple biomarkers have been tested to identify CIN lesions with a high risk of progression. P53, p16, and Ki67/Mib1 are amongst the best accepted for patient management [1]–[5]. It is known that proportions of proliferating cells increase with dysplastic stage. Recently, combined Mib1 and p16 analysis separated HGSILs based on progression risk [6]. Validation and acceptance of any biomarker requires an understanding of the molecular role that marker plays in disease. Others have analyzed the capacity of Mib1 expression to identify high risk lesions and assist in diagnosis of HGSIL. Some groups have developed algorithms to quantify the distribution of proliferating cells and have demonstrated the power of these quantitative features over conventional, subjective assessments [3], [5], [7]–[13].

It is well-accepted that smoking is a cofactor for development of CIN [14]–[27]. Diverse hypotheses attempt to explain the effects of smoking, however, while smoking is recognized as a CIN co-factor, the exact nature of interactions between smoking, HPV infection, and dysplasia remains unclear [18].

Herein, we report our analysis on the effects of HPV infection and smoking on cell proliferation for normal and neoplastic cervical epithelia. We sought to apply a rigorous semi-automated approach to quantify these effects in CIN lesions. To achieve this, we analyzed individual epithelial layers in a well-annotated, thoroughly reviewed patient cohort. Through this, we have gained insights into the impact of these factors on cell behavior for different disease stages. This work provides a rationale for wider evaluation of a combined approach involving clinical features (e.g. HPV, smoking status) and automated analysis of protein expression in epithelial layers as a biomarker for managing CIN.

## Materials and Methods

### Sample collection

Samples were chosen amongst 1850 patients (3735 biopsies) aged ≥18 that were collected during a multi-center study to evaluate Mib1 and p16 staining as a means of improving diagnosis of HGSIL [6]. Enrolled patients were those from a diagnostic population (i.e. had previously had an abnormal Pap test result). Seeking a distribution of lesion types and a cohort sufficiently large to power meaningful statistical analyses, we chose 196 patients for whom 453 biopsies were available. As this work sought to analyze the full thickness of tissue cell layers, we further restricted our sample cohort to those biopsy specimens with intact basement membranes and epithelial layers at pathology review (n = 261). Samples were chosen from those acquired at the British Columbia Cancer Agency (BCCA), one of four institutions in the wider study. All samples meeting the above criteria (n = 261) – including multiple samples from the same patient – underwent Mib1 staining and quantitative imaging (described below).

### Patient samples

Non-pregnant women ≥18 years were enrolled from 1999–2006. The study protocol was approved by the University of British Columbia/BC Cancer Agency Research Ethics Board (protocol #C02-0476) and written consent was obtained from all enrolled patients. Patient demographic features (Table 1) were obtained for all cases, with ethnicity categories defined based on US OMB criteria, age defined as the subject’s age at the time of the clinic visit, and smoking status defined based on current smoking habits. Regarding disease grade, biopsies were taken from one or two colposcopically abnormal areas and from two colposcopically-normal areas. Consensus diagnoses, based on review by expert pathologists, were used as the guiding criterion standard (see following section) [28].

**Table 1:** Demographic features for study patients.

Patient Demographics		n = 196
*Median Age (range)*		32 (18–66)
*Ethnicity*		
	White	143
	Black	2
	Hispanic	3
	Native American	4
	Asian	30
	Other	14
*Smoking status*		
	Current	55
	Former	46
	Never	95

### Consensus diagnoses

Histopathological specimens were reviewed at BCCA by both WHO and Bethesda criteria. Initial reviews were performed by one of the gynecological pathologists on clinical duty (the “pool”). There were seven pathologists in the pool, which also included study pathologists. A second blinded review was performed by one of two study pathologists. If the first and second readings agreed exactly in the WHO system, third reviews were not performed. Discrepancy of two grades based on WHO criteria mandated a third blinded reading by both study pathologists. Kappa scores for pathology readings have been reported [28]. In sum, for all diagnoses ranging from normal through CIN lesions to cancer, kappa-values ranged from 0.40 to 0.80; those in the HGSIL/cancer range were in the high 0.70–0.85 kappa-value range. While “biopsy diagnoses” described disease state for a given biopsy specimen, “patient diagnoses” described the highest grade disease detected by biopsy review in a patient (since multiple biopsies were obtained from each patient and different disease stages were sometimes identified).

### HPV testing

Endocervical samples were collected and tested for HPV using the Hybrid-Capture II system. This assay detects both low-risk (HPV-6, -11, -42, -43, -44) and high-risk (HPV-16, -18, -31, -33, -35, -39, -45, -51, -52, -56, -58, -59, -68) HPV types. All samples were processed using funds from public grants.

### Mib1 staining

Paraffin sections (4 µm) were mounted on slides, dried overnight (37°C), deparaffinised in xylenes, and rehydrated in alcohol. Next, endogenous peroxidase activity was blocked by 3% H_2_O_2_ in phosphate-buffered saline (PBS). Sections were immersed in sodium citrate buffer and microwave heated at power settings of 1000 W for 2 min, then 160 W for 15 min. Before immuno-staining, slides were soaked in PBS. Sections were incubated with biotinylated swine anti-rabbit antibody (DAKO) at 1∶100 for 30 min. Visualization of the complex was realized using diaminobenxidine/H_2_O_2_ for 10 min (room temperature). Two washes in PBS were performed before counterstaining with Mayer’s Hematoxylin. Sections were dehydrated using graded ethanol and mounted with a mixture of distyrene, plasticiser, and xylenes.

### Interpretation of immuno-histochemistry staining

Getafics [29], an in-house high-resolution image analysis system using a monochrome Charge Coupled Device (CCD) camera mounted on a light microscope and attached to an image analysis software package, acquired image data. The illumination wavelength was 600±5 nm, effective pixel sampling space within the sample plane was 0.34 µm^2^, and the effective pixel sampling area was 0.116 µm^2^. In each case, areas corresponding to diagnostic areas selected by study pathologists were carefully located by expert technicians. Images of that region were then captured. Regions of interest (ROI) were interactively and carefully delineated. Both basement membranes and superficial apical surfaces were separately defined.

A simple, automated thresholding method followed by an Otsu segmentation algorithm was applied to extract centers of gravity of all cell nuclei [30]. Each segmented image was revisited and manual correction was performed by cyto-technicians to identify nuclei “forgotten” by automated algorithms and to remove false-positive objects. Mib1-positive nuclei were interactively marked, with Mib1 status verified under microscope when necessary. Coordinates of the two membranes and spatial coordinates of the nuclei with negative/positive Mib1 staining results were then saved and used for mathematical analyses. Tangentially cut epithelia were removed from analysis.

### Voronoi diagrams, layers-based analyses, and Voronoi layer definition

Voronoi cell layers were defined thusly: all cells whose corresponding Voronoi polygons touched the basement membrane were assigned to layer 0 (basal layer). Layer 1 (parabasal layer) consisted of those cells which were not in layer 0 and had a neighbor in layer 0. Layer 2 consisted of those cells which were not in layer 1 or layer 0 and had a neighbor in layer 1. Higher number layers were defined similarly. Generally, layers from layer 2 to the penultimate layer observed were interpreted as “intermediate” epithelial layers, while final layers were termed the “superficial” epithelial layer.

**Figure 1:**
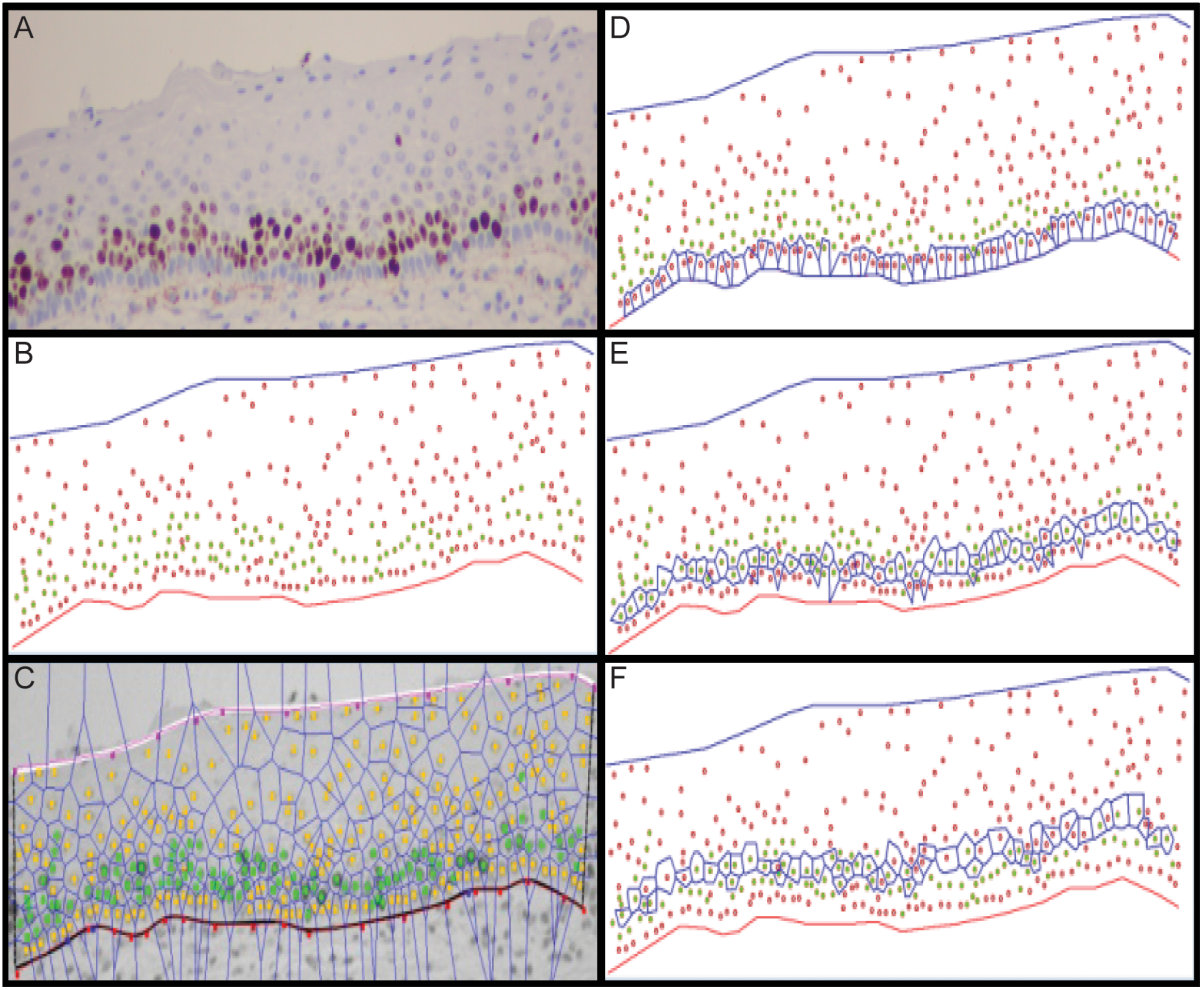
Spatial analysis of Mib1-stained cervical biopsies. We present: (A) an unprocessed, stained cervical biopsy cross section; (B) basal and superficial membranes that were first manually delineated by a technician through simple thresholding followed by automated segmentation of all nuclei, which generated candidate cell’s nuclear centers of gravity; (C) Mib1-positive nuclei that were identified manually by the technician (green dots) and Voronoi diagrams that were generated based on those centers of gravity; (D) the basal layer (bottom of panel) containing all nuclei whose associated Voronoi polygons intersected with the basal membrane; (E) layer 1 (the parabasal layer); and (F) successive layers that were incrementally calculated.

The percentage of Mib1-positive cells in each layer was calculated as the number of positive cells divided by the number of cells belonging in the layer. Voronoi diagrams and layers-based analyses were undertaken as follows: given a set of points S in a plane, a Voronoi tessellation for S is the partition of the plane which associates a polygon V(p) with each point p of S in such a way that all locations in V(p) are closer to p than to any other point in S [31]. Applied to centers of nuclei in the ROI, the Voronoi tessellation allowed each nucleus to be associated with a Voronoi polygon that could be viewed as its area of influence. In this way, the Voronoi diagram gives a very natural mathematical representation of the epithelium. With this diagram, it is straightforward to analyze the distribution of nuclei within a given region. We used structural features based on the Voronoi diagram and on Mib1 positivity to describe the spatial interrelations of Mib1-positive cells in conjunction with the tissue architecture. Given a finite set of points, the Voronoi polygons of those points on the outside edges of the set were unbounded (i.e. one vertex of those polygons will be located at infinity). The polygons corresponding to these points were called marginal polygons. For this reason, the intersection of a marginal polygon with the ROI (consisting of the basement membrane, the external membrane, and the lines joining the ends of these two membranes) was found. Each marginal polygon was replaced with the polygon resulting from the intersection of the marginal polygon with the ROI boundary. Additional details related to Voronoi tessellations are provided in Fig. 1.

### Statistical Analysis

All analyses were done with STATISTICA10. All p-values were two-sided, with a two-sided p-value<0.05 considered significant. For comparisons between groups, ANOVA testing was done. When testing showed a statistical difference across groups, Least Significant Differences testing or planned comparisons were used to compare groups two-by-two.

## Results

Two-hundred sixty-one biopsies obtained from this diagnostic population (i.e. cohort with a previous abnormal Pap test result) were stained with Mib1 (Table 2). Based on worst observed patient diagnosis in each case, 62% (24/39) of all “normal” samples were HPV-positive. Also based on the worst observed patient diagnosis, 61% (59/97) of Low-Grade squamous intraepithelial lesions (LGSILs, i.e. CIN1 lesions) were positive for oncogenic HPV, whereas 97% (122/126) of HGSILs (CIN2/3 lesions) were HPV-positive. Normal epithelia from cervices in which a HGSIL was found were on average thinner than epithelia from women in which no abnormalities were identified – and also from epithelia from women with LGSIL (data not shown). These differences were statistically significant (p = 0.04 and p = 0.02, respectively). There were no differences between the percentage of proliferating cells and the number of layers in these groups. Nevertheless, because of these differences in epithelium thickness, we restricted analyses to normal specimens from patients without abnormalities when describing normal tissues (Table 3). Hence, downstream analyses spanned i) all HGSIL biopsies, ii) all LGSIL biopsies, and iii) all “normal” biopsies from cervices of patients where no abnormality was detected in any biopsy.

**Table 2:** Results for Mib1-stained biopsy specimens (including stratification based on histopathological review of an individual biopsy specimen [“Biopsy Diagnosis”]; worst disease grade observed amongst multiple biopsies from a single patient [i.e. “Worst Patient Diagnosis”]; and infection status for High Risk HPV types [i.e. the 13 high risk types defined within the Hybrid Capture II system]).

Biopsy BeingInvestigated Diagnosis	Worst PatientDiagnosis	Negative for Hybrid CaptureII HPV high risk type	Positive for Hybrid CaptureII HPV high risk type	Total Number ofbiopsies studied
Normal	Normal	15	24	39
Normal	LSIL	28	43	71
Normal	HSIL	2	49	51
LSIL	LSIL	11	16	27
LSIL	HSIL	0	8	8
HSIL	HSIL	2	63	65
**Total**		**58**	**203**	**261**

**Table 3:** Mean value of epithelial thickness (µm), number of layers, and percentage of proliferating cells according to histopathological diagnosis (with standard deviations represented in parentheses.

PathologicalDiagnosis	Total Number	Patient Age	Thickness (µm)	Number ofLayers	% Proliferating Cells
Normal	39	37.6 (11.0)	420.7 (216.0)	9.9 (3.0)	15.1 (8.6)
CIN1	35	32.9 (8.9)	331.4 (196.0)	9.6 (3.5)	28.7 (17.2)
CIN2	31	30.3 (6.5)	334.2 (200.0)	11.0 (4.4)	34.3 (15.1)
CIN3	34	29.6 (7.5)	239.0 (125.0)	12.1 (5.4)	54.7 (19.7)

**Figure 2:**
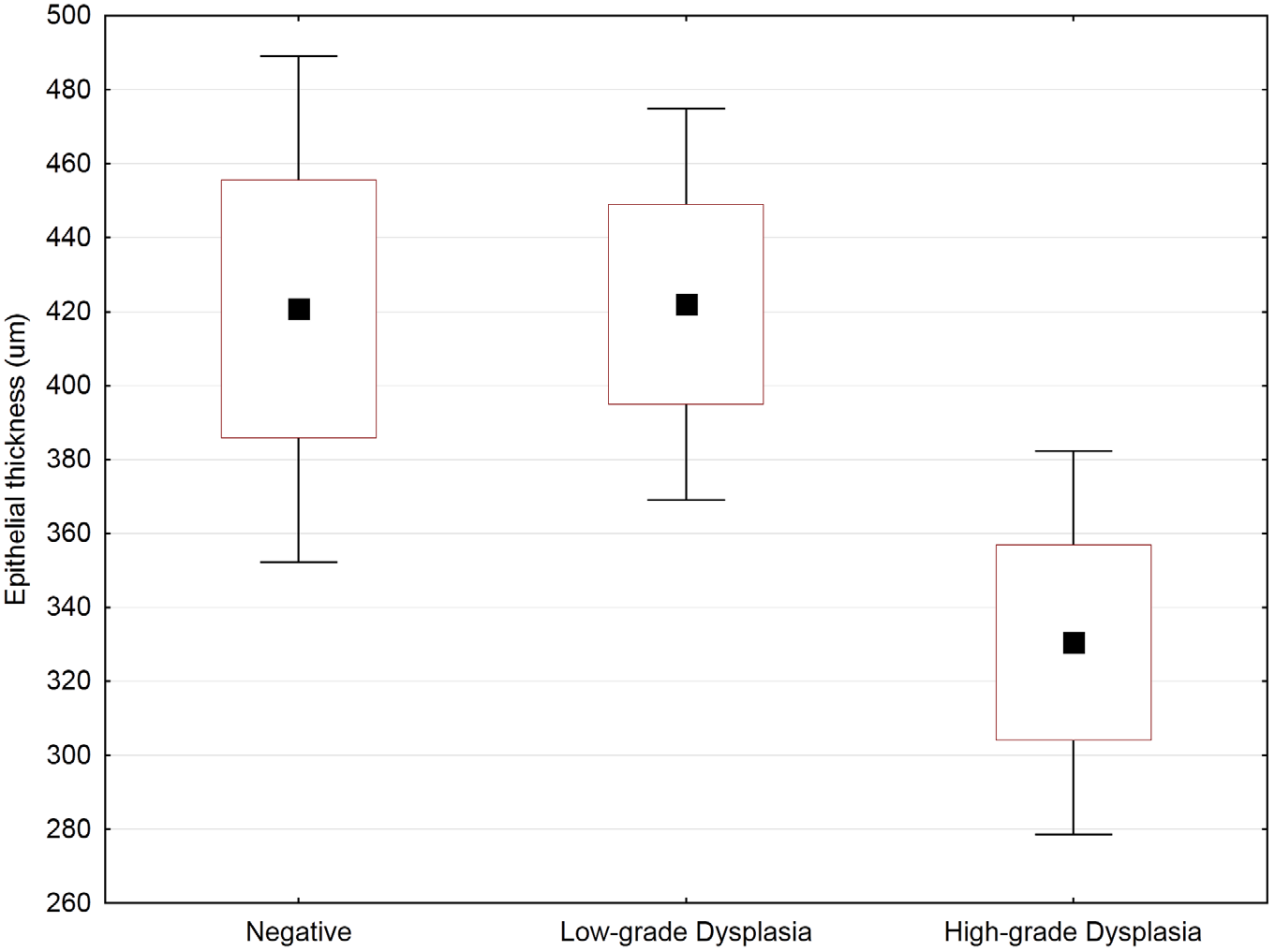
Epithelial thickness of normal epithelium in patients with no abnormalities, patients with LGSIL (i.e. CIN1), and patients with HGSIL (i.e. CIN2/3). No variability due to age was observed. The mid-point represents the median value; boxes represent the 25^th^ percentiles; and whiskers represent the 95th percentiles. Factorial ANOVA testing was followed by a *post hoc* Fisher LSD test for group-by-group comparisons, yielding: p = 0.97 for a normal vs. LGSIL comparison; p = 0.048 for a normal vs. HGSIL comparison; and p = 0.020 for a LGSIL vs. HGSIL comparison.

The mean and standard deviation of epithelial thickness, number of layers, and percentage of proliferating cells for four pathological grades were determined (Table 3). There was a consistent decrease in epithelial thickness from normal epithelium to CIN3 lesions. Epithelia of CIN1 lesions were on average 22% (89.3 µm) thinner than those of normal tissues. For CIN3 lesions, epithelia were 28% (95.2 µm) thinner than in CIN2 lesions. There was a statistically significant difference between normal and CIN3 tissues (p<0.0001). Additional epithelial thickness comparisons based on disease grade were undertaken (Fig. 2).

**Figure 3:**
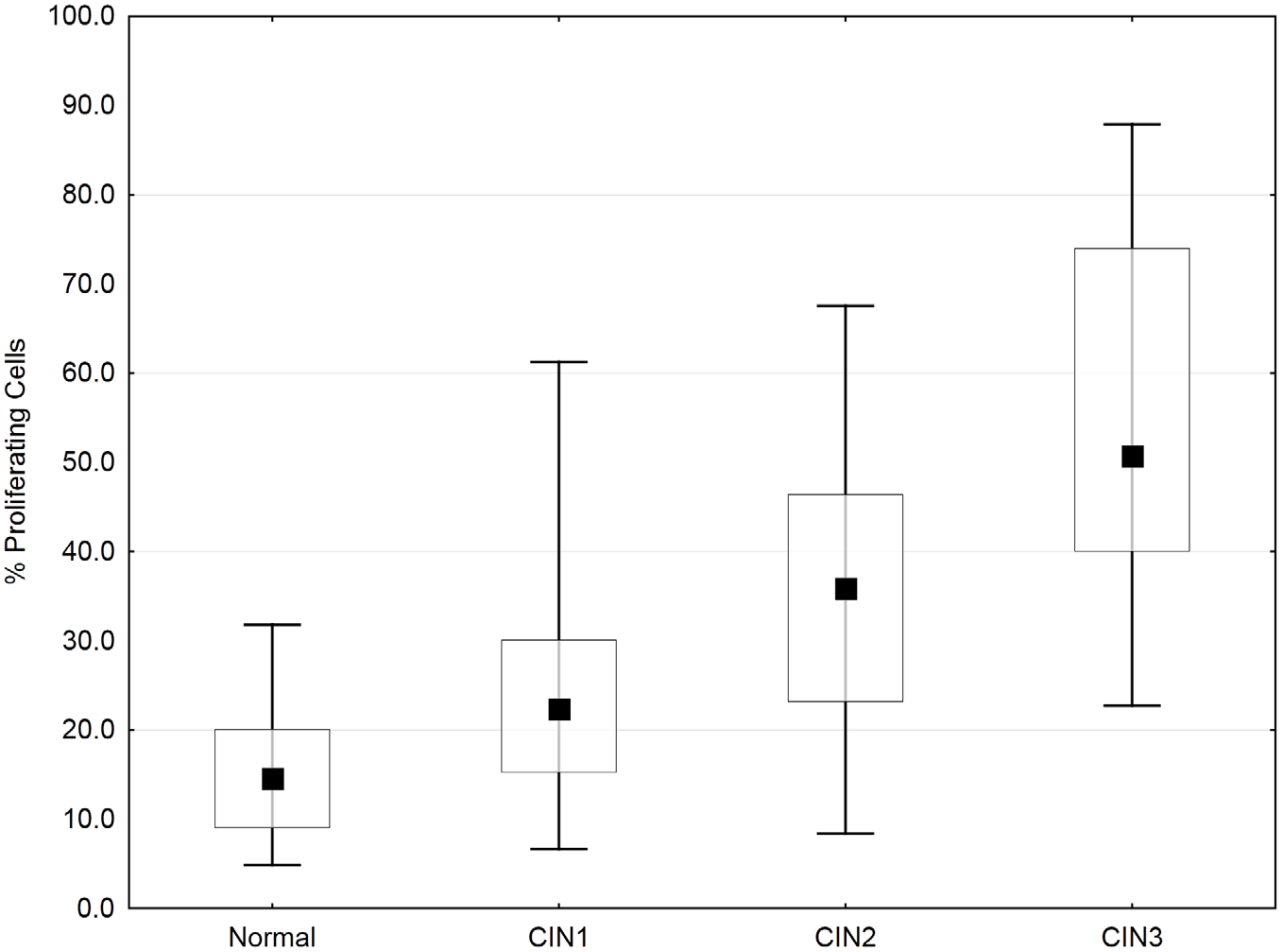
Proportion of Mib1-positive cells (i.e. proliferating cells) in the four diagnostic groups. The mid-point represents the median value; boxes represent the 25th percentiles; and whiskers represent the 95^th^ percentiles. Once again, factorial ANOVA testing was followed by a *post hoc* Fisher LSD test for group-by-group comparisons, yielding: p = 0.0006 for a normal vs. CIN1 comparison; p = 0.017 for a normal CIN1 vs. CIN2 comparison; and p = 0.00001 for a CIN2 vs. CIN3 comparison.

Voronoi layers were calculated as described above and in Fig. 1. The number of layers increased from normal to CIN3 tissues, with a statistically significant difference observed between CIN3 vs. normal specimens (p = 0.03) and between CIN3 vs. CIN1 lesions (p = 0.021) (Table 3). Fig. 3 compares the percentage of proliferating cells vs. disease grade. A clear trend shows increased percentages of proliferating cells vs. increased CIN grade. These increases were statistically significant for normal vs. CIN1 tissues (p<0.001) and for CIN2 vs. CIN3 lesions (p<0.00001).

### Layer-based cell proliferation and dysplasia

In normal epithelia, >60% of cell proliferation occurred in the parabasal layer and layer 2 (Table 4). Proliferation in the upper layers dropped rapidly to 14%, 8%, and 4% in layers 3, 4, and 5, respectively – and was almost negligible for higher layers. Interestingly, we observed a near-linear increase in the percentage of proliferating cells in both basal and parabasal layers from normal epithelium to CIN1, CIN1 to CIN2, and CIN2 to CIN3. The differentiation between CIN1 and CIN2 occurred in layer 3, where proliferating cells represented 50% of cells in CIN2 lesions vs. only 33% in CIN1 lesions. In higher layers, the proliferation profile of CIN1 lesions resembled the proliferation pattern of normal epithelial tissues. Contrarily, CIN2 lesions exhibited much higher proliferation rates, with 30–40% more proliferating cells in each higher layer.

**Table 4:** Percentage of proliferating cells – mean and (Standard Deviation) – in the first eight layers of cervical epithelium according to histopathology diagnosis.

PathologicalDiagnosis	layer 0 (basal)	layer 1 (para-basal)	layer 2	layer 3	layer 4	layer 5	layer 6	layer 7
Normal	5.9 (11.8)	32.9 (20.4)	31.3 (21.6)	15.6 (20.2)	7.8 (15.3)	3.4 (9.8)	1.8 (5.8)	2.2 (6.5)
CIN1	21.5 (21.0)	43.5 (26.2)	39.6 (24.6)	26.0 (25.2)	17.4 (22.3)	9.5 (17.8)	9.8 (19.1)	14.0 (23.2)
CIN2	20.8 (19.5)	46.5 (19.7)	49.9 (23.8)	37.7 (25.1)	36.7 (28.3)	30.9 (28.0)	24.2 (26.9)	33.0 (31.1)
CIN3	34.8 (24.4)	58.6 (22.7)	62.7 (20.0)	64.5 (24.8)	57.6 (27.6)	55.0 (25.0)	51.1 (28.4)	58.3 (24.1)

Detailed methods for delineating epithelial layers are found in Materials and Methods, Fig. 1.

**Figure 4:**
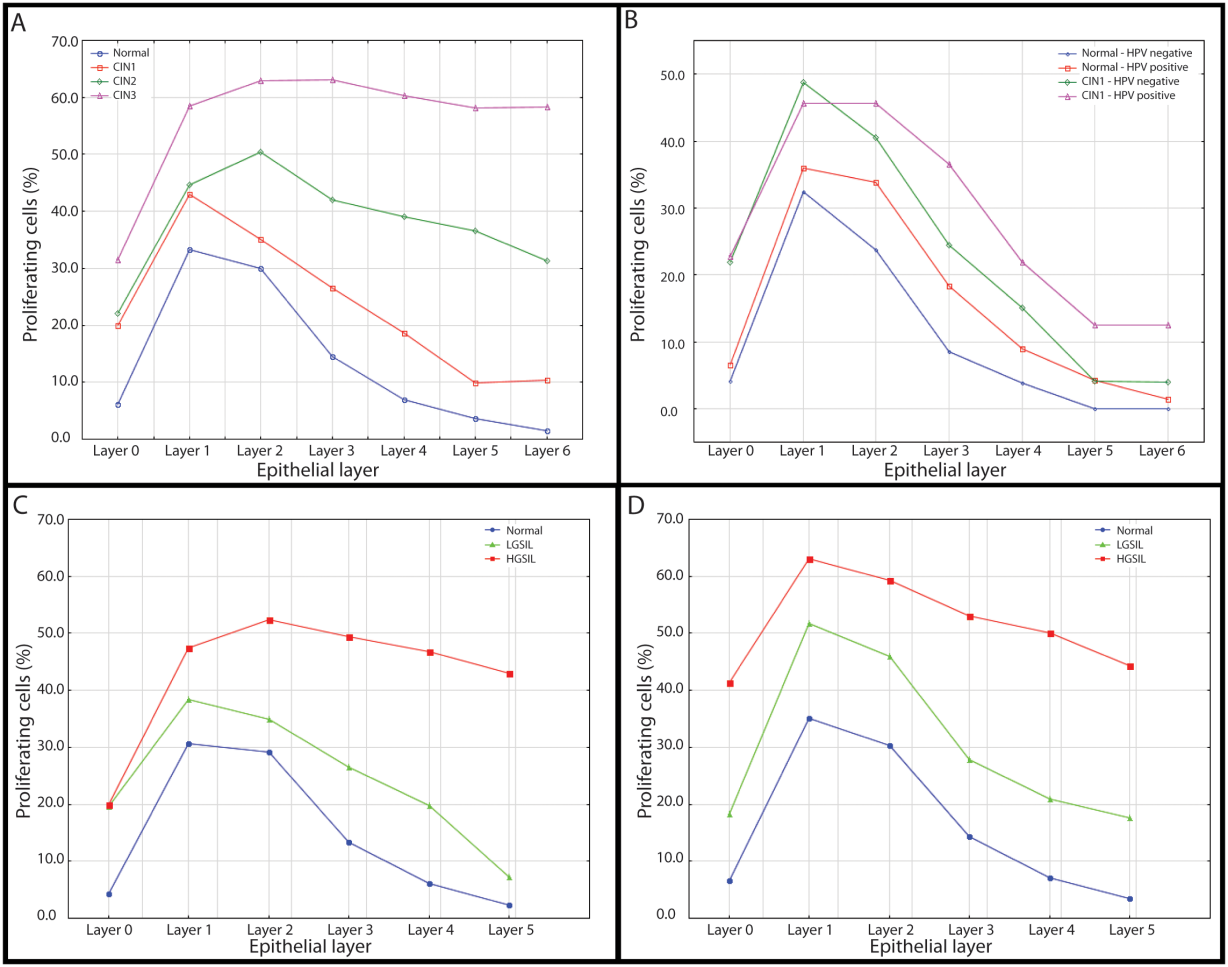
Average percentage of proliferating cells in the first layers of cervical epithelium. In all panels, layers 0 and 1 represent the basal and parabasal layers, respectively. A) Percentage of proliferating cells in the first seven layers of cervical epithelium as assessed for each pathology grade. B) Percentage of proliferating cells in the first seven layers of cervical epithelium for normal and CIN1 tissues based on high-risk HPV type infection status. C) Percentage of proliferating cells in the first six layers of epithelium of non-smokers, where LGSILs represent CIN1 lesions and HGSILs represent CIN2/3 lesions. D) Same representation as C), only results presented are for patients with smoking histories.

We next returned to assessing proliferation on a layer-by-layer basis for the four diagnostic groups (Fig. 4A). The percentage of proliferating cells in the basal layer was much higher in CIN3 lesions vs. normal epithelial tissues (30% vs. 6%) and almost identical between CIN1 and CIN2 lesions (∼22%). A similar pattern was observed in the parabasal layer (layer 1): 60% of CIN3 nuclei were Mib1-positive vs. only 33% for normal cases. CIN1 and CIN2 lesions exhibited similar percentages of Mib1-positive nuclei (∼44%). For each of the three CIN groups, we noted that the percentage of Mib1-positive nuclei in the parabasal layer was approximately double the percentage in the basal layer: 30% vs. 60% for CIN3 cases and 20% vs. 40% for CIN1 and CIN2 cases. In normal epithelia, the difference was larger: 33% of Mib1-positive nuclei were in the parabasal layer vs. 7% in the basal layer.

This relative proliferation pattern changed in upper layers. The percentage of Mib1-positive nuclei for CIN1 cases decreased by ∼10% from layer 1 to 2 but increased by ∼6% from layer 1 to 2 in CIN2 lesions. The decrease in the percentage of Mib1-positive nuclei in CIN2 and CIN1 lesions was almost identical from layer 2 to layer 6, with an 18% decrease in CIN2 lesions and 14% decrease in CIN1 lesions, respectively.

### Layer-based cell proliferation and dysplasia and HPV infection

We noted several HPV impacts on proliferation patterns (Fig. 4B). This analysis was restricted to normal tissues and CIN1 lesions (too few HPV-negative HGSILs were available for meaningful analyses related to infection). We observed similar stratifications of proliferation profiles for normal and CIN1 groups based on HPV status; for both normal and CIN1 cases, a higher percentage of Mib1-positive nuclei were noted in layer 3 for HPV-positive cases vs. HPV-negative ones. The layer proliferation profile of CIN1 HPV-positive tissues resembled the layer proliferation profile of the CIN2 lesions. (Proliferation profiles for women ≤40 vs. >40 years old showed near identical results, confirming absence of an age effect on cell proliferation rates [data not shown]).

### Layer-based cell Proliferation and dysplasia and smoking status

**Figure 5:**
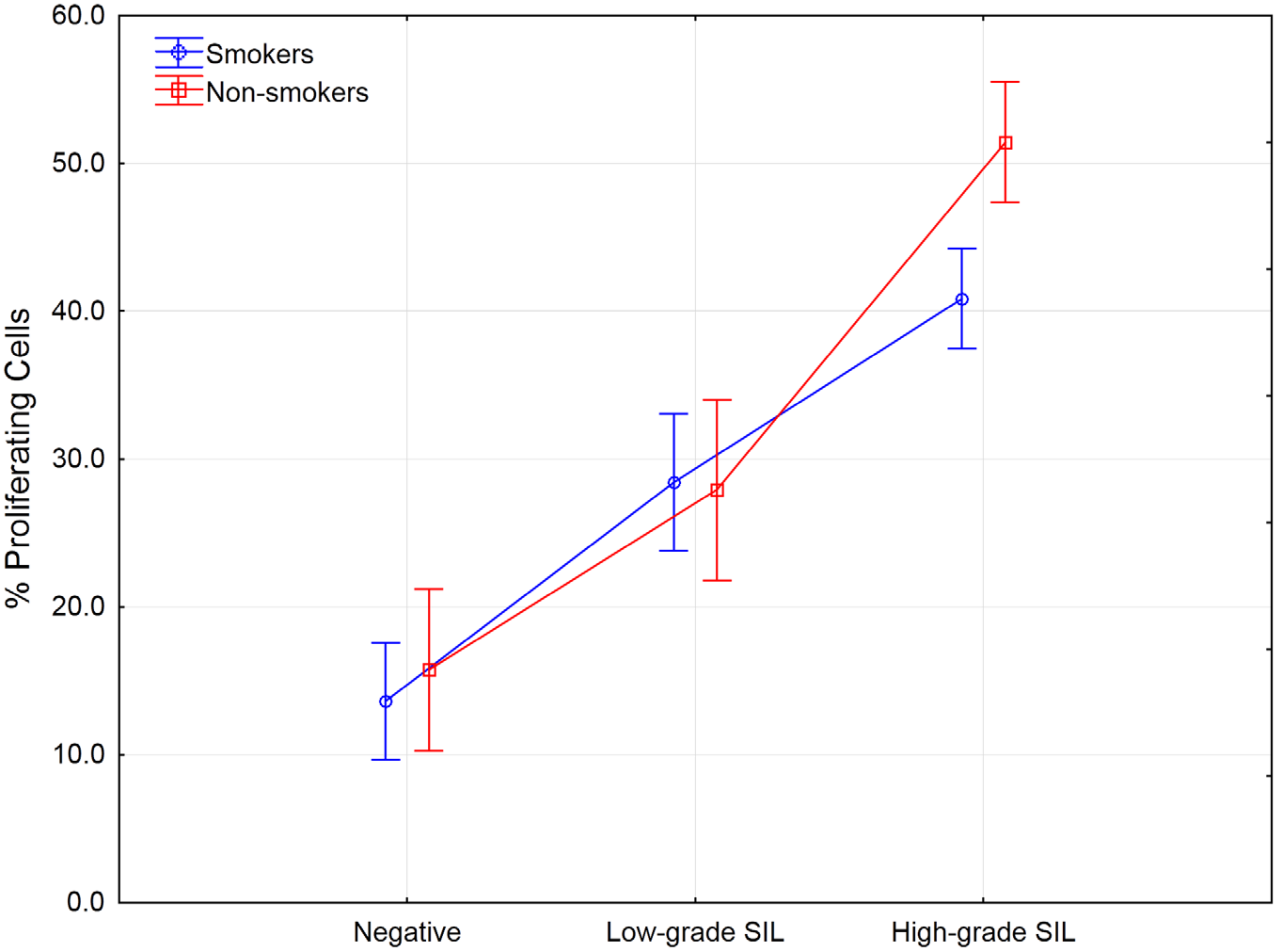
Percentage of proliferating cells in the full thickness of the epithelium for normal epithelium, LGSILs, and HGSILs for non-smokers and smokers. Factorial ANOVA/*post hoc* Fisher LSD testing revealed the following results when comparing results from non-smokers vs. smokers: for cases negative for disease, p = 0.75; for LGSILs, p = 0.94; and for HGSILs, p = 0.049.

Fig. 5 shows the impact of smoking status on cell proliferation rates when the epithelium was analyzed at full thickness. Limited separation was observed between non-diseased and LGSIL tissues. The difference for HGSILs between smokers vs. non-smokers was not statistically significant. Fig. 4C and D shows proliferation profiles for the first 6 layers of normal, LGSIL, and HGSIL cases for non-smokers and smokers, respectively. Overall cell proliferation was detected as higher for smokers for all layers. The most marked difference between smokers vs. non-smokers occurred in the basal layer of HGSILs, where the percentage of proliferating cells doubled for smokers (19% vs. 40%, p<0.001).

## Discussion

Novel methods described herein allowed quantification of cell proliferation on a per-epithelial-layer basis (Fig. 1). This yielded insight into the spatial effects of smoking, HPV infection, and dysplastic processes in cell proliferation for CIN lesions. Combined analysis of all epithelial layers revealed an increase in cell proliferation with progression through dysplastic stages, agreeing with previous work (Fig. 3, Table 4) [1], [12]. On average, the percentage of proliferating cells increased with progression: from 16% in normal epithelia to 27% in CIN1, 36% in CIN2, and 55% in CIN3 (Fig. 3). This increase in proliferating cells was accompanied by a decrease in epithelial thickness through loss of differentiated cells that were replaced by newly dividing, smaller cells (Fig. 2, Table 3). Consequently, cell density increased together with the number of cell layers. While proliferation rates for various CIN stages were comparable to those of previous reports, we observed an elevated proliferation rate in normal tissues [32]. This result could have been influenced by whether or not “histologically normal tissues” were taken from both diseased and non-diseased patients and also by general variability in disease state classification for LGSILs vs. normal tissues.

One key observation was that differences in proliferation rates between CIN1 and CIN2 lesions occurred in specific epithelial layers (Fig. 4A). While proliferation rates for these lesions were nearly identical for basal and parabasal layers, proliferation rates in CIN2 lesions were ≥50% higher in all higher layers. Whereas combined analysis of all epithelial layers of CIN1 and CIN2 lesions is not sufficient to discriminate these disease stages, this result suggests that honing analysis within epithelial layers above basal/parabasal layers may yield more accurate clinical grading (which would impact estimation of disease progression risk). Increased proliferation rates in higher epithelial layers for CIN2 lesions (vs. CIN1 lesions) may be driven by the impacts of greater viral genome integration (e.g. disruption of viral E2 expression), which is associated with disease progression [33]. Interestingly, while no difference in basal layer proliferation rates was observed for CIN1 vs. CIN2 lesions, we noted a marked increase in basal layer proliferation rates for CIN3 lesions. This increase may be attributable to increased disease signalling in higher epithelial layers, a feedback mechanism that may further disease progression.

We also observed HPV-driven proliferation rate differences within a given disease stage. Moreover, these differences were detected in specific epithelial layers. Given high prevalence of HPV infection in advanced disease stages, we were only able to derive statistically meaningful results related to HPV status for normal and CIN1 tissues. For both these tissue types, we noted higher rates of proliferation for HPV-positive cases relative to HPV-negative cases in epithelial layers above basal and parabasal layers (Fig. 4B). These data suggest that analysis of individual epithelial layers may be an effective, independent means of determining infection status/disease stage – and that assessing cell proliferation rates in each epithelial layer may be a useful tool for guiding disease management decisions. These results also suggest that CIN1 HPV-negative lesions may be driven by a hyperplastic response that up-regulates proliferation of basal cells – and that daughter cells subsequently respond in the same fashion (which would suggest that they in turn follow the same control sequence). In HPV-positive cases, it may be that HPV infection disrupts this control sequence, resulting in a different pattern of proliferation (vs. non-infected tissues). The molecular mechanisms by which higher proliferation rates in HPV-positive normal and CIN1 lesions occur only in epithelial levels beyond the basal and parabasal layers remains unknown. Follow-up analysis of a larger panel of HPV-positive/-negative CIN2 lesions is needed to determine whether proliferation in higher epithelial layers can delineate more advanced disease stages.

Finally, we noticed marked smoking-mediated differences in the distribution of proliferating cells in CIN lesions. When epithelia were analyzed at full thickness, negligible separation in the percentage of proliferating cells was observed for normal and LGSIL tissues from smoking vs. non-smoking patients (a small degree of separation was observed for HGSILs, with smoking cases exhibiting somewhat higher percentages of proliferating cells) (Fig. 5). Nonetheless, separation into individual epithelial layers revealed doubled proliferation rates in the basal layer of HGSILs from smokers vs. non-smokers (along with increased proliferation rates in the parabasal layer) (Fig. 4C, D). This may be indicative of smoking-related factors driving cells out of senescence, which could in turn result in a larger pool of cells harboring molecular alterations, increasing the likelihood that a given cell in the tissue field will mutate further and progress to later stage disease. Interestingly, we also noted significantly higher proliferation rates in the parabasal layer of smokers vs. non-smokers for LGSILs but no significant difference in basal layer proliferation for these cases. The striking difference in basal layer proliferation rates for HGSILs may be attributable to inflammation effects due to smoking, which can in general be associated with increased cell proliferation in other tissue types [34]–[36]. The absence of this smoking-driven phenomenon in LGSILs may suggest that the degree of inflammation in these cases is insufficient to trigger higher proliferation rates. Our finding of no major differences in normal tissue cell proliferation rates for smokers vs. non-smokers agrees with previous reports [37]. Taken together, our data suggest a strong effect of smoking on cell proliferation that manifests independently of disease stage and most prominently in basal and parabasal epithelial layers.

## Conclusion

Our analyses of cell proliferation in normal cervical and CIN tissues have identified epithelial layer-specific features that delineate disease grades. Our results also show that proliferation within cell layers varies depending on HPV infection and smoking status. We have demonstrated the utility of computer-assisted histopathological slide analysis for identifying novel cell features associated with disease grade. More accurate clinical diagnoses based on this technique could improve patient management by facilitating more accurate estimations of disease progression risk. This work bolsters the argument that research into HPV and smoking impacts on cell proliferation must account for spatial differences in proliferation based on individual epithelial layers (instead of relying on analyses of the entire epithelium at its full thickness). Independent analyses using the approaches described here are needed to determine whether cell proliferation in epithelial layers beyond the basal and parabasal layers has utility for discriminating CIN1 and CIN2 lesions. Further analyses are also needed to determine the biological processes causing the HPV- and smoking-driven cell proliferation changes we observe within epithelial layers.
